# Supplementary material for: Addition of Polyphenols to Drugs: The Potential of Controlling “Inflammaging” and Fibrosis in Human Senescent Lung Fibroblasts In Vitro
Source: Int J Mol Sci. 2024 Jun 28;25(13):7163. doi: 10.3390/ijms25137163 (PMC11241747; doi:10.3390/ijms25137163)
Supplement: Supplementary file 1 [file ijms-25-07163-s001.zip › ijms-3069479-supplementary.pdf]

Supplementary material*

Manuscript: "Addition of polyphenols to drugs: the potential of controlling "inflammaging" and fibrosis in human senescent lung fibroblasts in vitro."

Results

The higher doses of nintedanib and pirfenidone assessed were cytotoxic. The dose of 1 μM of nintedanib and 0.8 mM of pirfenidone were chosen for subsequent experiments (Figure S1).

The combination of nintedanib with chlorogenic acid and epicatechin, as the combination of pirfenidone with caffeic acid, chlorogenic acid, epicatechin, quercetin, and resveratrol were able to reduce the cell viability of control MRC-5 cells, but not in senescent MRC-5 cells (Figure S2).

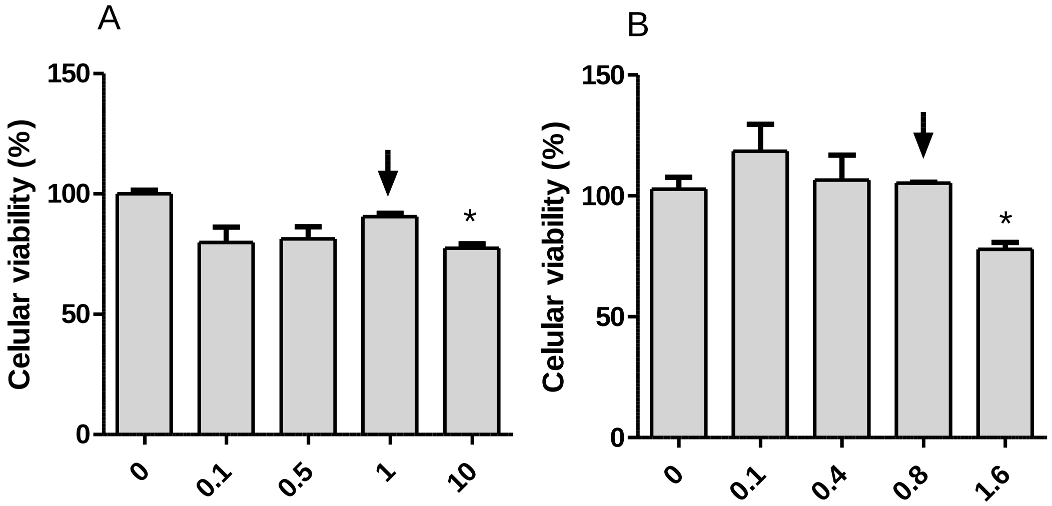

Figure S1. Cytotoxicity evaluated in control MRC-5 cells incubated for 24 h with a concentration range of the drugs Nintedanib (0.1-10 μM, **A**) and Pirfenidone (0.1-1.6 mM, **B**). The experiment was carried out in triplicate with one repetition. * $p<0.05$ when compared to nontreated MRC-5 cells. The arrows show the concentration chosen for each drug for subsequent assays.

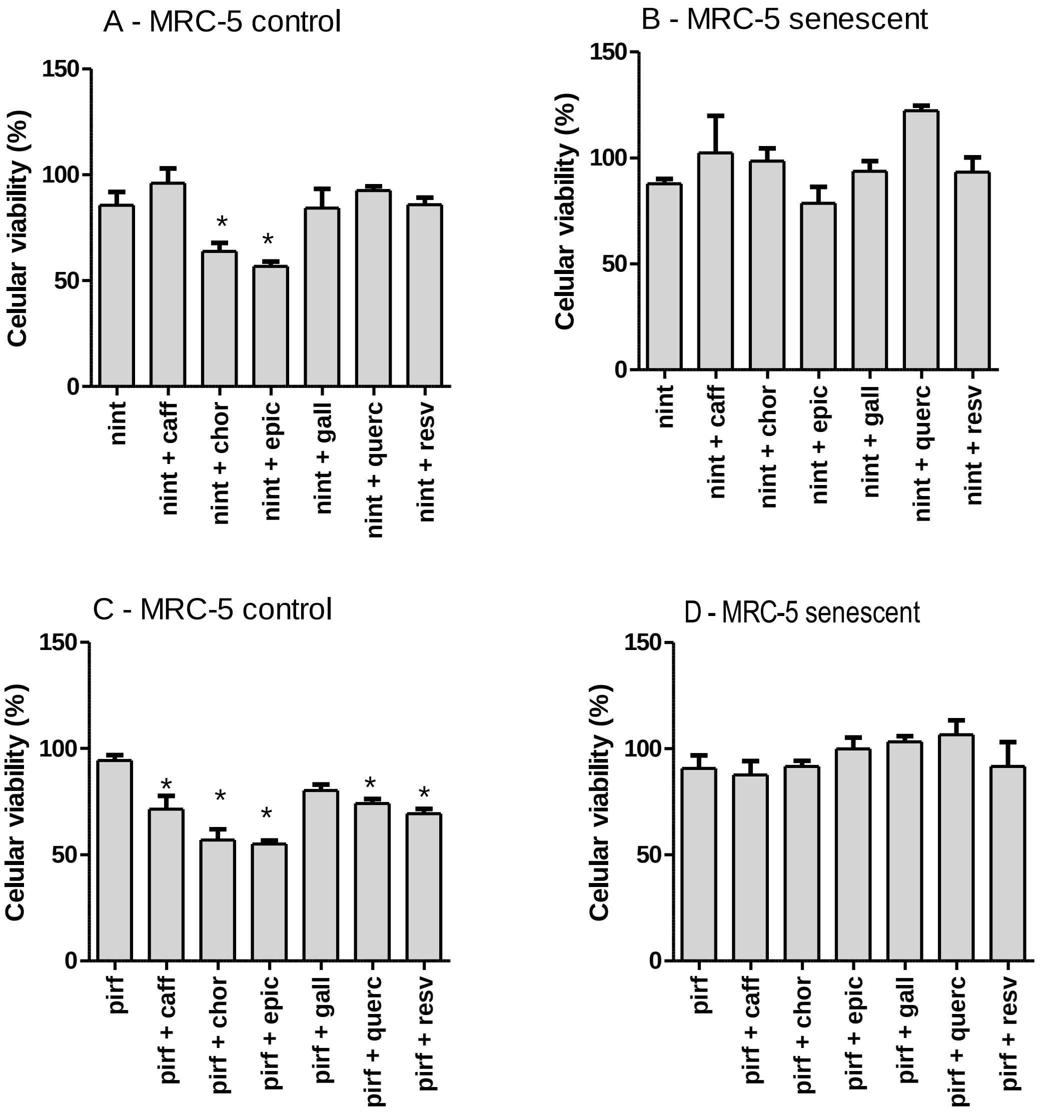

Figure S2. Cytotoxicity evaluated in control (**A, C**) or senescent (**B, D**) MRC-5 cells incubated for 24 h with a nintedanib (nint; 1μM) and pirfenidone (pirf; 0.8 mM) in combination with caffeic acid (Caff; 1000 μM), chlorogenic acid (1000 μM), epicatechin (Epic; 1000 μM), gallic acid (Gall; 100 μM), quercetin (Quer; 100 μM) or resveratrol (Resv; 100 μM). The experiment was carried out in triplicate with one repetition. * $p<0.05$ when compared to nontreated MRC-5 cells.

The doses of drugs and epicatechin employed in epithelial-mesenchymal transition (EMT) assays did not present cytotoxicity in A549 cells (Figure S3).

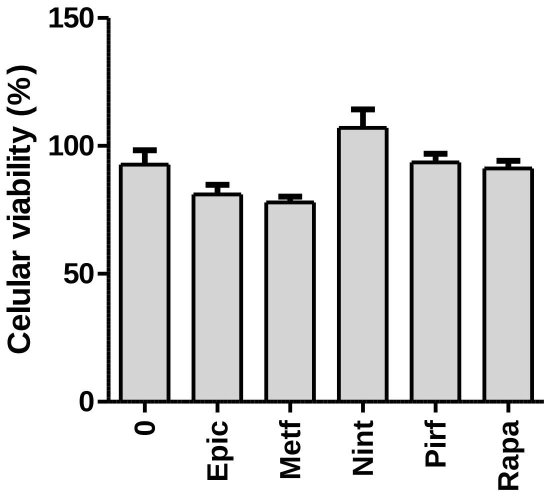

Figure S3. Cytotoxicity evaluated in control A549 cells incubated for 72 h with a concentration range of the epicatechin epicatechin (Epic; 100 μM), metformin (Metf; 1 mM), nintedanib (Nint; 1 μM), pirfenidone (Pirf; 0.8 mM) or rapamycin (Rapa; 10 nM). The experiment was carried out in triplicate with one repetition. * $p<0.05$ when compared to nontreated MRC-5 cells. The arrows show the concentration chosen for each drug for subsequent assays.

Methods

Table S1: Primer's sequence

Gene	Forward	Reverse
CDKN2A	GAGCAGCATGGAGCCTTC	CGTAACTATTCGGTGCGTTG
CDKN1A	TCACTGTCTTGTACCCTTGTGC	GGCGTTTGGAGTGGTAGAAA
COL1A	TGA CGA GAC CAA GAA CTG CC	GCA CCA TCA TTT CCA CGA GC
COL3A1	AAGTCAAGGAGAAAGTGGTCG	CTCGTTCTCCATTCTTACCAGG
ACTA1	ATG CTC CCA GGG CTG TTT TC	CTT TTG CTC TGT GCT TCG TC
CDH1	CGAGAGCTACACGTTCACGG	GGGTGTCGAGGGAAAAATAGG
b-actin	CCAACCGCGAGAAGATGA	CCAGAGGCGTACAGGGATAG

* Additional supplementary data:
Pharmaceuticals 2024, 17, 70. https://doi.org/10.3390/ph17010070
